# Microbiome science of human excrement composting

**DOI:** 10.1093/ismejo/wrae228

**Published:** 2024-11-09

**Authors:** Jeff Meilander, J Gregory Caporaso

**Affiliations:** Department of Biological Sciences, Northern Arizona University, Flagstaff, AZ 86011, United States; Pathogen and Microbiome Institute, Northern Arizona University, Flagstaff, AZ 86011, United States; Department of Biological Sciences, Northern Arizona University, Flagstaff, AZ 86011, United States; Pathogen and Microbiome Institute, Northern Arizona University, Flagstaff, AZ 86011, United States

**Keywords:** human excrement, compost, 16 s, amplicon, manure, biosolids, microbiome, latrines, compost toilet, soil, sustainability

## Abstract

Linear waste management systems are unsustainable and contribute to environmental degradation, economic inequity, and health disparities. Among the array of environmental challenges stemming from anthropogenic impacts, the management of human excrement (human feces and urine) stands as a significant concern. Over two billion people do not have access to adequate sanitation, signifying a global public health crisis. Composting is the microbial biotechnology aimed at cycling organic waste, including human excrement, for improved public health, agricultural productivity and safety, and environmental sustainability. Applications of modern microbiome omics and related technologies have the capacity to support continued advances in composting science and praxis. In this article, we review literature focused on applications of microbiome technologies to study composting systems and reactions. The studies we survey generally fall into the categories of animal manure composting, biosolids composting, and human excrement composting. We review experiments utilizing microbiome technologies to investigate strategies for enhancing pathogen suppression and accelerating the biodegradation of organic matter. Additionally, we explore studies focused on the bioengineering potential of microbes as inoculants to facilitate degradation of toxins, such as pharmaceuticals or per- and polyfluoroalkyl substances. The findings from these studies underscore the importance of advancing our understanding of composting processes through the integration of emerging microbiome omics technologies. We conclude that work to-date has demonstrated exciting basic and applied science potential from studying compost microbiomes, with promising implications for enhancing global environmental sustainability and public health.

## Introduction

Exponential human population growth has profoundly disrupted Earth’s natural cycles, resulting in climate change, biodiversity loss, and widespread pollution. The escalating scarcity of landfill space [1] driven by vast quantities of organic waste generated by modern throwaway societies [2], presents a monumental environmental challenge. This issue is further compounded by the growing volume of livestock manure, which has surged over the past two decades [3], contributing to environmental pollution and greenhouse gas emissions [4]. Composting offers a viable solution by transforming these organic materials into nutrient-rich products, thus mitigating their environmental impact.

Among this array of challenges, the management of human excrement (HE; defined here as human feces and urine), stands as a massive concern due to the considerable volume produced globally (373 billion kg per year [5]) and its toxicity if not properly managed. Access to adequate sanitation infrastructure varies widely across the world [6], and in areas where properly functioning infrastructure exists, the process uses exorbitant amounts of energy [7].

Prior to treatment, each flush of a traditional toilet expels 7.5–23 liters of potable water along with valuable nutrients found in HE into sewers, where it mixes with contaminants from storm water runoff and industrial waste before reaching wastewater treatment plants (WWTP) [8]. These contaminants include heavy metals, pharmaceuticals, polychlorinated biphenyls, oils, fertilizers, pesticides, and “forever chemicals”, such as per- and polyfluoroalkyl substances (PFAS) as well as fecal indicators and pathogens from untreated HE and animal manure [9, 10].

This cocktail of nutrients and contaminants requires diverse chemical, biological, and physical treatments. Treated wastewater is discharged into nearby water bodies and biosolids—stabilized organic materials—can be applied to soil for remediation or agriculture. However, eliminating persistent pollutants from treated wastewater and biosolids remains a challenge [11] as residues like PFAS can contaminate soils and groundwater, disrupt ecosystems through endocrine disruption [12], and bioaccumulate in living organisms [13].

**Figure 1:**
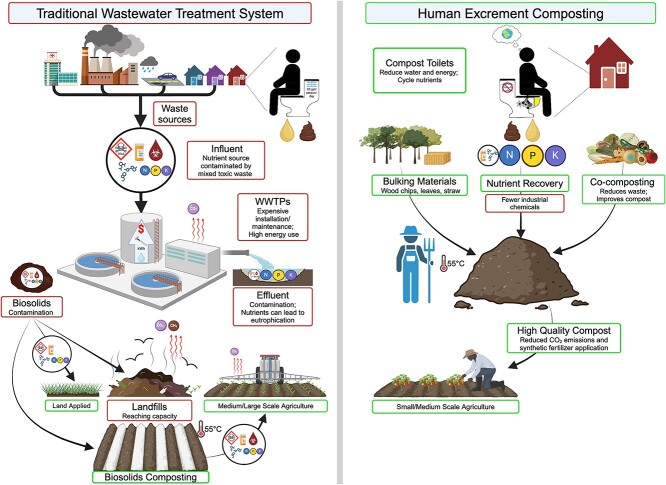
Traditional wastewater treatment systems versus HEC. Properly managed HEC can provide many benefits over traditional wastewater management systems for managing HE. In HEC with CTs, when the CT reaches capacity the material must be transferred to an actively managed compost pile. Effective composting begins with a balanced mixture of HE and BM, such as sawdust, straw, or wood chips. BM provides structure, absorbs excess moisture, and creates air pockets that enhance aerobic conditions, which are crucial for efficient microbial decomposition. Co-composting with kitchen scraps improves compost quality and reduces organic waste production. During the thermophilic phase, where temperatures can exceed 55°C, a succession of microbial communities degrades organic matter and significantly reduces pathogen levels. Thermophilic composting of HE has also been shown to degrade various pharmaceuticals present in HE. This process allows up to 91% of N, 83% of P, and 59% of K in HE to be safely recovered. If the resulting material is used as an agricultural soil amendment, this can reduce reliance on synthetic fertilizers and enhance soil fertility and crop yields. Created in BioRender.

Composting toilets (CTs) offer a decentralized approach to HE management that contrasts sharply with traditional biosolids composting. By avoiding the introduction of HE into the wastewater stream at the source, CTs reduce the burden on WWTPs, thereby decreasing water and energy-intensive treatment processes and avoiding contamination with industrial wastes (Fig. 1). HE treatment can be achieved through HE composting (HEC), a human-managed and microbially-driven process resulting in a safe, nutrient-rich, humus-like product [14]. The recovery and reuse of key nutrients in HE—specifically up to 91% of nitrogen (N), 83% of phosphorus (P), and 59% of potassium (K)—can effectively reduce reliance on synthetic fertilizers [15], thus enhancing soil fertility and agricultural yields [16]. CTs require minimal infrastructure (plumbing), and require little to no freshwater, making them considerably cheaper and easier to install than conventional systems [17], especially in areas where traditional wastewater infrastructure is nonfunctional or nonexistent including research field sites, eco-villages, national parks (including backcountry areas without running water), rural communities, and low- and middle-income countries.

CTs can also offer desperately needed public health benefits. Two billion people worldwide are estimated to lack access to adequate sanitation facilities and 450 million are limited to practicing open defecation. In both cases, exposure to pathogens is significant [6], and could be reduced through the use of properly managed CTs [18, 19]. This exposure contributes to diarrheal diseases, which stand as the second leading cause of mortality among children under five years of age (>1200 deaths per day globally) [20].

CTs offer sustainable approaches to nutrient management, however, to fully harness the potential of composting, we must improve current understanding of HEC, particularly regarding the microbial dynamics within these systems. For example, *Pseudomonas* has been identified as a beneficial organism in biosolids production at WWTPs, municipal scale food waste composting, and HEC. Despite recent and widespread application of microbiome profiling and multi-omics technologies to study diverse habitats, including diverse composting and waste treatment systems [21], there remains a gap in their application to HEC research.

Composting methods and sanitation practices vary widely across different socioeconomic and geographical contexts, influenced by resource availability and infrastructure. In low-income regions, particularly in parts of sub-Saharan Africa and South Asia, open defecation remains prevalent due to limited sanitation infrastructure. In contrast, lower- and middle-income rural areas often utilize septic tanks for on-site waste management, as seen in parts of rural Australia and the US. Meanwhile, CTs are popular in eco-conscious and remote communities, such as those in certain areas of Sweden and Nepal where sustainable nutrient cycling is prioritized and where conventional sewage systems are challenging to install. For high-income urban areas, advanced WWTPs, such as those in Singapore and Berlin, support efficient, centralized wastewater management, with an emphasis on nutrient recovery and environmental safety. This diversity in sanitation practices reflects the impact of economic and infrastructural factors on waste management solutions, showcasing a range of approaches that meet different communities’ needs to varying extents.

In this review, we begin by defining composting and briefly outlining the process, referencing relevant composting literature. We then illustrate insights gained from high-throughput sequencing and other omics technologies in various composting and waste treatment methods, including in manure and biosolids composting. We review select studies of non-HEC systems, even though this review focuses on HEC, because similar composting principles apply to different types of organic materials, so those findings can aid in identifying and further investigating beneficial microbes for potential applications in HEC operations. We conclude by advocating for the need to apply these advanced technologies to bolster HEC research, highlighting areas where these technologies could be applied to advance our understanding and praxis of HEC to catalyze advancements in sustainable waste management practices and improvements in global public health.

### Composting science

We have incorporated elements from various perspectives [14, 22] to define *composting* here as: the human-directed, microbially driven process of aerobic biodegradation of organic wastes at temperatures between 45 and 70°C, yielding a soil amendment that is safe, mature (high degree of process completion), stable (resistant to further decomposition), and nutrient-rich. If the composting process remains <45°C, it is classified as “mesophilic composting”. Although mesophilic composting does not achieve the same rate or efficiency of pathogen reduction and nutrient biodegradation, prolonged treatment times have demonstrated effectiveness in ensuring safety [18]. However, specific parameters leading to safe products therein remain less well understood [19].

**Figure 2:**
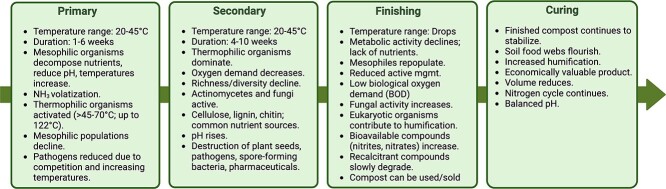
Composting phases. The flow chart illustrates the four distinct phases of composting, each characterized by specific temperature ranges and processes. Composting is commonly divided into phases to better understand the biological and chemical transformations that occur during decomposition, where phases are defined based on temperature changes. [112]. Created in BioRender.

Composting is a well-characterized process (Fig. 2) relying on compost operators to administer parameters such as carbon-to-N ratios (C:N), moisture, pH, and oxygen content [14] throughout four distinct phases. “Feedstocks” are the organic, foundational components of compost (e.g. food waste or manure) that are mixed with bulking material (BM; e.g. leaf litter or wood chips) to create the microbial environment supporting preferred ranges of composting parameters (Table 1). Proper management stimulates microbial activities, elevating temperatures into the “thermophilic range” where biodegradation and pathogen reduction is optimal [18]. The World Health Organization recommends that HEC reach 55°C or higher for between 7 and 30 days followed by a 2–4 month curing phase where the compost fails to reheat when turned thus promoting decomposition through the reintroduction of fungi and actinomycetes, and to allow for further pathogen reduction, [23]. Under 40 C.F.R. § Part 503, the US EPA similarly requires temperatures above 55°C for at least 3 days when producing class A biosolids—treated sewage sludge that meets strict pathogen reduction and contaminant standards, making it safe for general land application, including in public areas and food production.

**Table 1:** Optimal parameters and key variables for effective composting. A summary of the essential compost variables and their preferred ranges for achieving effective composting. Each variable plays a critical role in the composting process. Adapted from [113].

Variable	Description	Preferred range
Feedstocks	Organic materials collected for composting (e.g. kitchen scraps, landscaping waste, manure).	
BMs	Combined with feedstocks to optimize microbial activity and improve compost texture.	Combined with feedstocks to achieve preferred ranges.
Carbon to N ratio (C:N)	Ratio of carbon to N (C:N) in compost feedstock.	25:1–40:1
Moisture	Water content in the compost pile.	50%–60%
Oxygen content	Adequate oxygen availability within pore spaces for aerobic decomposition. Proper aeration helps maintain oxygen.	>10%
Temperature	Optimal temperatures to achieve proper sanitization.	50–65°C
pH	Acidity or alkalinity of the compost.	6.5–8.0
Bulk density	The weight of compost per unit volume—maintains proper aeration and porosity.	400–600 kg/m^3^
Qualitative variables	Color, odor, and texture serve as quick quality indicators. Attention to other variables is crucial.	

Amplicon sequencing methods including ribosomal ribonucleic acid (rRNA) and ITS profiling, have increasingly been applied to study microbiomes of composting systems [24, 25]. Integrating other omics technologies—for example, to identify active genes and metabolic processes—can enable bioprospecting from compost systems with the potential to uncover enzymes, antibiotics, and other bioactive compounds with biotechnological and/or biomedical applications [26]. The microbial diversity inherent to composting represents an untapped resource for bioengineering initiatives aimed at enhancing specific microbial functions, such as the degradation of recalcitrant compounds, the biosynthesis of biofertilizers [27], and the production of biosurfactants [28]. Additionally, the identification and validation of biomarkers of compost maturity—including specific microbial taxa, enzymatic activities, and metabolite profiles—enable more precise and reliable assessments of compost quality, thereby optimizing its effectiveness as a soil amendment [29]. This convergence of bioprospecting, bioengineering, and biomarker discovery paves the way for advancing the sustainability and efficiency of composting in the context of HEC and beyond.

In the next sections we review literature on the applications of microbiome-focused multi-omics technologies in HE and more general composting research. An overview of the specific technologies used in the studies that we review are provided (Table S1).

### Studying composting with microbiome science technologies

Microbes drive the composting process. Their relative abundances, interactions, and successional patterns are influenced by compost variables, recipes, phases, and substrates [30], as well as more general factors including resource availability [31], inter- and intraspecific competition [32], and abiotic and biotic factors [33].

Specific taxonomic groups of bacteria (e.g. *Bacillales* and *Clostridiales*) and fungi (e.g. *Eurotiales* and *Glomerellales*) dominate compost microbiomes at different phases leading to a dynamic interaction between microbial populations and composting conditions [33]. Experiments have documented phasic and temporal responses between fungal and bacterial communities during pig manure composting analogous to those seen during forest restoration [34] as well as differences in microbiome composition as a result of specific plant species [35] and total available organic material [36]. Furthermore, microhabitats within compost systems differ spatially, temporally, and in response to changes in pH, temperature, or byproducts secreted by other organisms in the community [22, 37].

Because deoxyribonucleic acid (DNA) sequencing and other omics technologies identify precise patterns of microbial succession, reduce biases arising from culture-dependent research [38], and provide unprecedented insight into microbial activity, they drive the acquisition of new knowledge throughout the composting process [39]. This information can be applied to identify specific bioindicators of compost phases or phase changes [29], or of the degradation of toxins, pathogens, and pharmaceuticals. Ultimately, this can facilitate better (and potentially fully automated) compost management by enabling microbiological- and data-driven adjustments to temperature, aeration, and moisture that optimize microbial activity for effective decomposition.

The use of microorganisms by humans, for example in fermentation, energy production [40], and other bioengineering and biotechnological advancements [41], has a history pre-dating our awareness of the existence of microorganisms. Microbiome science technologies, such as the cornerstone technique of 16S rRNA gene sequencing, have opened new doors in this domain by providing a pathway to the rational design or optimization of these applications [42]. We now turn to review research into the under-explored microbial communities driving composting of animal manure, biosolids, and HE. Ultimately, we believe that this knowledge can be applied to HEC and CTs to promote safer and more sustainable waste management.

### Manure composting

In concentrated animal feeding operations (CAFOs), where a certain number of animal units are raised in confined situations for an extended period of time, manure is often stored in lagoons.

This practice poses risks such as a rupture of the lagoon walls and leaching of N and P into nearby water sources [4], thus leading to eutrophication, spreading antibiotic resistance genes (ARGs) into the environment [43] and contaminating drinking water [44] inducing adverse health effects, such as cyanosis in infants [45].

Generally, there are fewer restrictions on the use of manure in agriculture compared to biosolids or raw HE. Nutrients within manure can be reclaimed through composting or direct land application [46], but pathogens such as *Salmonella*, *E. coli*, *Listeria*, *Staphylococcus*, *Klebsiella*, *Enterobacter*, *Serratia*, and *Campylobacter* in manure pose safety concerns. To mitigate these pathogens, “processes to further reduce pathogens” found in 40 CFR (Code of Federal Regulations) Part 503 recommends that facilities utilize composting above 55°C, heat drying, irradiation, or pasteurization [47].

Although not composting based on the definition we provided in this manuscript, there was an observed increase in microbial diversity and biomass in soils amended with cattle manure [48], likely due to the higher levels of total organic carbon and total N reported in the soil following the amendment [49]. Amplicon-based detection revealed the immediate reduction of manure-associated bacteria, but spore forming organisms, such as *Clostridium*, *Paeniclostridium*, *Romboutsia*, and *Turicibacter* persisted nearly a year later. Ensuring thermophilic temperatures for at least three days or adding chemical agents, such as peracetic acid [50], urea [51], or fine coal gasification slag [52] can reduce pathogenic bacteria, viruses, and bacteriophages, offering potentially useful treatments of manure prior to land application. Manure management regulations vary by country and state, but generally focusing on the identification of pathogens or fecal indicator bacteria, crop nutrient needs, application timing, and environmental protection [53–55].

Recalcitrant compounds such as lignocellulose pose challenges for composting efficiency and economics. Inoculating with specific bacteria, such as *Kocuria*, *Microbacterium*, *Acidovorax*, and *Comamonas*, could enhance compost quality and efficiency [56]. During co-composting of dairy manure and sugarcane leaves, an inoculant containing the thermophilic bacteria *Ureibacillus suwonensis*, *Geobacillus thermodenitrificans*, and *Bacillus licheniformis*, known to produce biosurfactants capable of increasing composting efficiency [28], was associated with a prolonged thermophilic phase and increased rate of organic matter degradation [57]. In a different study [58], the addition of maize straw to chicken manure composting increased the abundance of thermophilic bacteria, such as *Limnochordaceae*, *Planifilum*, *Oceanobacillus*, and *Thermobifida* and accelerated the rate of early stage microbial succession compared to composting chicken manure alone.

In the early phases of pig manure composting, the bacterial taxa *Bacillales* and *Clostridiales* predominated, supported by abundant cellulose content. Subsequently, as the composting process advanced, fungal taxa such as *Eurotiales* and *Glomerellales* proliferated, correlating with the accumulation of recalcitrant compounds left behind as metabolic byproducts of bacterial decomposition thus contributing to enhanced humification and further biodegradation [33].

By leveraging amplicon sequencing, research teams aim to further enhance manure composting efficiency [59], which holds potential for more sustainable nutrient cycling and waste management in agriculture [60]. Given the similarities in microbial processes between composting animal manure and HE, findings from manure composting studies can offer valuable insights for advancing HEC practices and optimizing nutrient recycling and waste management in agricultural contexts.

### Biosolids composting

Biosolids, a byproduct of WWT, can be disposed of through various disposal methods including landfilling, composting, incineration, and land application. Meanwhile, treated wastewater can be reintroduced into receiving water bodies or marketed as reclaimed water [61]. The chemical attributes of biosolids are significantly influenced by diverse waste sources and treatment methods, such as aerobic digestion, liming, or composting. These methods impact their microbial compositions, pathogen abundance, and suitability for agricultural applications [62]. Composted biosolids must undergo various processing techniques and meet strict regulatory standards regarding pathogens, heavy metals, and nutrient content before they can be reused.

Annually, global biosolids production exceeds >33 million dry tons [63] and represents a valuable nutrient source [64] typically containing between 50%–90% of their N composition in organic compounds [65]. Biosolids also contain essential micronutrients such as B, Cl, Cu, Fe, Mn, Mo, and Zn which are crucial for plant growth but often lacking in conventional chemical fertilizers [66], and offer the potential to recover elements, such as Ag, Cu, Au, P, Fe, Pd, Mn, Zn, Ir, Al, Cd, Ti, Ga, and Cr [67]. However, concerns regarding the land application of biosolids stem from the origin and composition of the influents through the contents of the effluents, which can contain pathogens, heavy metals, ARGs, PFAS, and endocrine disrupting compounds.

Tracking microbial succession through the WWT process is essential for understanding the effectiveness of treatment methods, assessing potential risks to public health and the environment, and informing management practices to ensure the safety and sustainability of these systems. For example, seasonality and influent sources changed the biofilm formation in aerobic granular sludge treatment plants [68] in dewatered biosolids from a WWTP in Colombia [69]. Additionally, *Pseudomonas* spp. have been shown to tolerate elevated heavy metal concentrations and degrade xenobiotic pollutants and therefore could provide insight into bioengineering these traits [70].

An observed shift from methanogens to sulfur-metabolizing and surfactant-degrading bacteria in an anaerobic membrane bioreactor (AnMBR) [71] demonstrates the adaptability of microbial communities to challenges such as surfactant and sulfate presence in wastewater. Surfactant accumulation can impair treatment performance, so by supporting bacteria capable of degrading surfactants and metabolizing sulfur, the AnMBR enhances its ability to manage complex wastewater compositions, improve treatment efficiency, and support sustainable water recycling. This technology’s effectiveness in degrading organic pollutants and recovering nutrients, along with its low energy consumption, makes it suitable for controlled ecological life support systems in space missions, thereby advancing space exploration capabilities.

The adaptability of microbial communities in treatment systems, as seen in AnMBRs underscores the potential for targeted microbial functions to enhance treatment outcomes across varied wastewater contexts. One study assessed the bacterial diversity in various stages of treatment from a WWTP in Hong Kong [72]. In this system, seawater is used in toilet flushing and thus raises the salinity of the influent. Researchers observed that activated sludge samples, utilized during aerobic WWT, represented a species-rich environment. The microbial composition of digestion sludge exhibited distinct characteristics relative to a freshwater sludge digester, including low evenness dominated by a high abundance of *Kosmotoga* spp. which may be attributed to the incorporation of seawater.

The versatility of microbial communities can also facilitate in identifying pathogen persistence within biosolids, a crucial factor for ensuring the safety and effectiveness of treatment processes and the responsible reuse or disposal of biosolid materials. A previous study characterized pathogen diversity in biosolids subjected to mesophilic and thermophilic anaerobic digestion and composting [73]. Their study consistently identified *Mycobacteria* and *Clostridia* across all samples, including opportunistic pathogens *Mycobacterium fortuitum*, *Mycobacterium phlei*, and *Mycobacterium chelonae*. In Ireland, a study utilized polymerase chain reaction (PCR) to determine that autothermal thermophilic aerobic digestion (ATAD), a tertiary treatment process for sewage, was capable of reducing pathogen and fecal coliform loads [74]. At a WWTP in Berlin, Germany, researchers reported increased levels of *Legionella* and *Leptospira* in effluent when compared to the influent [75]. If the effluent is not properly depleted of human pathogens, nutrient (C and N) loading from the effluent can increase pathogen abundance in the environment [76] and contribute to their residence in soil and water habitats [77]. However, enteric bacteria such as *Campylobacter* and *Salmonella* spp. were undetectable in WWTP effluent and decreases in *Acinetobacter*, *Aeromonas*, and *Pseudomonas* were observed in another study [75].

In a previous study [78] *Devosia* and *Bradyrhizobium* genera, commonly associated with plant-growth promotion (PGP) and phytohormone production, were found in freshly dewatered and 4-year-old, stockpiled biosolids. Additionally, *Bacillus* spp. were linked to the degradation of lignin and lignocellulose producing phenolic and quinone compounds. These compounds are crucial precursors for humus formation, and promote the maturation process in biosolids composting [79]. Furthermore, fungal communities were found in mesophilic and thermophilic anaerobic digesters that likely catabolize organic matter, providing a valuable resource for archaeal and bacterial communities [80]. Other metabolic byproducts, such as volatile fatty acids may serve as potential nutrients for microbial groups [81].

During the thermophilic phase of ATAD, biodegradation of organic material resulted in a reduction in the activity of denitrifying and nitrifying bacteria likely attributed to an elevated pH [74]. *Desulfobacterales*, crucial in N cycling within mangrove forests [82], was prevalent in the influent of a WWTP [72] and holds potential for targeted utilization to enhance N cycling efficiency thereby potentially offsetting the decrease in denitrifying and nitrifying bacterial activity. Other nutrient cycling bacteria such as *Comamonas denitrificans* (present in biofilms of activated sludge), *Nitrospira* spp*.* (nitrite oxidation), and *Simplicispira limi* (associated with enhanced biological P removal (EBPR)) to mitigate eutrophication [83] were identified in WWTP effluent [75].

As previously observed, [74] increased levels of DNase activity, likely bolstered by abundant extracellular DNA stemming from cell lysis induced by thermophilic temperatures. This extracellular DNA could serve as a valuable nutrient source for other microbes within the community or as a vector for horizontal gene transfer (HGT) [84]. ATAD and thermophilic composting degrades exogenous DNA and therefore could mitigate antibiotic resistance through HGT, enhancing the safety of biosolids prior to their land application [74].

### Human excrement composting

Launched in 2015 as part of the 2030 Agenda for Sustainable Development, the UN Sustainable Development Goals provide a global framework to tackle pressing issues like poverty, inequality, climate change, and environmental degradation, aiming for a sustainable and equitable future. Goal 6 specifically seeks to advance sustainable water and sanitation management by ensuring universal access to safe water, sanitation, and hygiene, while improving water quality and protecting aquatic ecosystems [85]. Many organizations are contributing to this effort by addressing open defecation and expanding access to improved sanitation facilities, including CTs.

HE composting can take place in CTs or on varying scales, from small backyard systems to large municipal facilities, following general composting protocols. (Fig. 1 and Table 1). Human fecal contamination poses major public health and environmental risks, particularly in surface waters. Identifying contamination sources by environmental regulatory agencies is challenging, as *E. coli*, *Enterococcus* spp., *Bacteroidales*, or *Clostridium* spp. detections can stem from various origins, including municipal waste spills, agricultural runoff, and wildlife. Contamination levels are typically assessed through standard culturing, quantitative PCR (qPCR), and the detection of human-associated microorganisms like *Bifidobacterium adolescentis*, *Bifidobacterium dentium*, and human enterovirus, which are strong indicators of human fecal contamination [86]. The US EPA regulations outline qPCR protocols to identify fecal indicator bacteria in recreational waters [87] as qPCR offers a rapid turnaround time, delivering results within a few hours and allowing for timely decision-making.

In a study conducted at a HEC facility in Haiti operated by the non-profit organization SOIL [24] a PhyloChip microarray was utilized to assess the effectiveness of thermophilic composting in reducing fecal microbiota and pathogenic organisms below detectable limits [24]. This organization collects HE and produces bagged compost for donation or sale to the community, underscoring the importance of reducing exposure to fecal bacteria and pathogens, particularly in high-risk areas. The results demonstrated that typical fecal bacteria such as *Prevotella*, *Lachnospiraceae*, and *Escherichia*, as well as opportunistic pathogens like *Salmonella* spp., were reduced below detection limits. Despite an overall decrease in microbial richness and abundance during the thermophilic phase, the genera *Thermobifida, Bacillus,* and *Geobacillus* increased in abundance, consistent with findings from other composting studies [78].

### Night soil

For centuries, agrarian societies disposed of HE directly onto agricultural lands [88]. Later referred to as night soil, it was often collected and transported under the cover of darkness due to the widespread aversion to visibly handling and transporting the excrement [89]. As civilizations expanded, waste removal systems became essential to removing HE from burgeoning city centers, and HE was typically directed toward agricultural fields or bodies of water [90]. Cultural, social, economic, and political influences reinforced the importance of recycling night soil, but rapid population growth, increases in population density, and industrialization substantially increased volumes of HE that needed to be managed [91]. Eventually, transporting night soil to surrounding agricultural areas became cost prohibitive, exacerbating the already formidable management challenges.

In many regions globally, the use of night soil compost (NSC) in agriculture is still practiced [25] there are ongoing concerns regarding its safety when applied to soils due to potential pathogen presence. Prior to their application, thermophilic temperatures during composting can lead to a significant reduction in opportunistic pathogens [18]. However, in areas where dry toilets are used, these temperatures are usually not achieved [19].

Despite challenges with managing the HE, dry CTs offer extremely compelling benefits. They provide accessibility for those without toilets, they effectively reduce odor through dehydration, and they have minimal infrastructure requirements. They are also easy to maintain, and can create economic opportunities for those installing these systems, and for those collecting the material for reuse [92]. Additionally, when the HE is properly managed (e.g. by composting) they can help prevent contamination of water and soils [93].

A recent study [25] assessed the microbial community dynamics, pathogenic risks, and phytotoxicity of NSC from the northwestern region of Himalayas. Their findings indicate that the NSC produced met the US EPA criteria for class A compost, exhibiting safe levels of pathogens. Major genera found in the NSC were associated with cellulose degradation, N fixation, and plant-growth-promoting traits. Although ARGs were detected in bacterial strains raising concerns for NSC reuse, the finished product was deemed non-harmful toward plant roots. This study also revealed that NSC shared predominant phyla when compared to HE, cattle manure, food waste compost, vermicompost, and activated sludge, but at the genus level NSC was distinct from these groups.

16S rRNA gene sequencing was applied to samples collected over a 30 day period from a microflush CT designed for the Ghana Sustainable Aid Project [92]. Initially, the bacterial community resembled that of the human gut, however, by day 4, the microbiome of the filter-digester bed began transitioning toward taxa commonly associated with environmental sources. The liquid effluent from the toilet was treated with solar disinfection and slow sand filtration (SSF). SSF appeared to introduce new taxa, possibly originating from the soils.

Unlike the Himalayan study [25], an investigation of dry CTs in Mexico evaluated the effectiveness in reducing fecal coliforms but only 36% of toilets achieved class A compost status after 6 months [94]. In this study, desiccation was the primary mechanism for fecal coliform reduction rather than biodegradation. Drier samples with greater solar exposure resulted in a higher proportion of class A samples.

In a study conducted in the Kathmandu Valley, Nepal, it was found that *Clostridium perfringens* exhibited the highest resistance among bacterial indicators in urine-diverting dehydrating toilet units, where wood ash was a primary additive [95]. The elevated pH from the addition of ash reduced microbial degradation rates and therefore heat production decreased [18]. When environmental conditions are unfavorable, *C. perfringens* forms endospores and its presence may be attributed to the existence of small microhabitats harboring favorable environmental conditions. The failure of these toilets to attain thermophilic temperatures may have also fostered regrowth.

In a separate investigation [96] the psychrotrophic bacterium *Glutamicibacter arilaitensis* LJH19 was identified in NSC. Through whole-genome sequencing, they identified its enzymatic capabilities, demonstrating amylase, cellulase, and xylanase activities even at low temperature (10°C) suggesting a mechanism by which biodegradation could occur in mesophilic composting. Furthermore, this organism exhibited PGP characteristics and evidence of siderophore production, a trait with potential applications in various fields including bioremediation, biocontrol of plant pathogens, and the development of unique antimicrobial therapies [97].

### Pit latrines

In low- and middle-income countries, onsite sanitation technologies including pit latrines, which collect HE in a hole in the ground, serve >1.8 billion people. Pit latrines contaminate groundwater, and financial constraints force the poorest users to manually empty the waste or neglect maintenance, leading to environmental contamination and exposure to fecal pathogens [98].

Sampling these pits for waste-based epidemiology also presents substantial occupational hazards to public health professionals. Researchers sampled various depths of pit latrines in Malawi to identify the most effective method for assessing pathogen levels, revealing no significant differences in pathogen detection at different depths [99]. Surface sampling of latrines could yield representative data, offering insights into community health and at the same time minimizing exposure risks for public health professionals.

Using the same data [99], another research group [100] characterized the microbial populations at various depths in pit toilets across different geographical regions in Malawi. Their analysis revealed a dominance of fermentative bacteria, specifically *Clostridium sensu stricto 1*, across all depths and locations, with taxa associated with methanogenesis pathways exhibiting increased prevalence with greater depth. The microbiomes in pit latrines were distinct from those found in activated sludge, municipal sewage sludge anaerobic digesters, and the human gut. Their findings suggest that microbial community structure and metabolic processes in pit latrines are shaped by consistent environmental conditions, rather than geographic location or waste characteristics, with microbial processes potentially specialized for conditions like varying oxygen levels, nutrient availability, and waste composition.

Understanding these distinctions is crucial for optimizing waste management practices, as strategies effective in activated sludge or anaerobic digesters may not be directly applicable to pit latrines, and vice versa. This could influence the design of interventions aimed at improving sanitation and reducing associated health risks. We hypothesize that microbiome changes and microbial succession from feces-like to environmental microbiomes occur much more slowly in pit latrines than in well-managed CTs [24, 25, 92]. Adding a temporal component to future pit latrine microbiome studies would allow testing this hypothesis and comparing the microbial safety of both approaches.

### Application of multi-omics tools to further improve sustainable waste management

Both qPCR and amplicon sequencing have identified microbial succession patterns, effective pathogen reduction strategies, and key microbial taxa involved in inoculation, competition, PGP, and ARG reduction. However, advanced techniques like metagenomics, metatranscriptomics, and metaproteomics offer deeper insights into these ecosystems, detecting the presence of pathogens and ARGs, and unearthing enzymatic biodegradation and metabolic activities occurring within waste microbiomes. As these methods become more cost-effective, they hold promise for advancing sustainable waste management. This section reviews the limited application of these technologies in HEC and their potential benefits based on their use in other waste management systems.

During composting, cellulose serves as a vital carbon source. A metaproteomics analysis of agricultural waste mixed with cow manure demonstrated that bacterial communities, dominated by *Thermobifida*, appeared to be primarily responsible for cellulose degradation, and fungal communities, dominated by *Thermomyces* and *Aspergillus*, were associated with hemicellulose degradation [101]. Additionally, β-glucosidase (BGL), an enzyme that degrades cellulose into glucose, was produced by fungi in municipal compost systems [102]. Metatranscriptomics revealed that microbial communities producing BGL showed differential expression of glucose-tolerant and non-glucose-tolerant BGL genes, likely adapting to fluctuating glucose levels during composting through diverse regulatory strategies [103].

Carbohydrate metabolism was identified as the principal metabolic pathway of Actinobacteria, Bacilli, Alphaproteobacteria, Betaproteobacteria, and Gammaproteobacteria classes during municipal composting [102]. In the same study, an enzyme for N fixation was found likely to be produced only by *Rhodobacter sphaeroides*, and an enzyme for denitrification only by *Pseudomonas stutzeri*, illustrating the ability to associate specific microbial activity with particular organisms: a precursor to rational inoculant development. Similarly, the identification of genes involved in methanogenesis [69] could improve methane gas capture from agro-industrial wastes, potentially supporting renewable energy development [104].

Further investigations using metagenomics have uncovered a wide variety of microorganisms and their functions in composting systems, highlighting both benefits and potential risks. For example, metagenomic studies identified DNA bacteriophages in vermicompost (composting with worms), alongside a high abundance of *Aeromonas*, a pathogen associated with gastrointestinal infections in humans [105]. More recent research has detected genes related to ARGs and heavy metal resistance in animal manure and NSC [25, 106].

Metagenomics analyses of wastewater treatment (WWT) systems has identified microbial communities capable of degrading polycyclic aromatic hydrocarbons (PAHs) found in pollutants like coal and crude oil, with some genes linked to *Pseudomonas thermotolerans* and *Mycobacterium hassiacum* actively expressing ARGs, as revealed through metatranscriptomics [107, 108]. These insights underscore the benefits of thermophilic composting, which can help reduce environmental and health risks associated with biosolid applications, as regulated under the USDA National Organics Program [55].

Several researchers applied metagenomics and metatranscriptomics to samples from a large-scale composting operation within the São Paulo Zoo Park in Brazil, and found that the decomposition of recalcitrant lignocellulose was primarily facilitated by bacterial enzymes from the Clostridiales and Actinomycetales orders [109]. Their results also suggested that cellulose and hemicellulose degradation were likely performed by cellulosomal enzymes, known for their efficiency in breaking down complex plant polysaccharides. Metagenomics resulted in the near-complete reconstruction of five bacterial genomes associated with biomass-degrading environments and a biodegrading bacterial species, potentially representing a new genus within the Bacillales order [21].

Metatranscriptomics data allowed the researchers to reclassify the composting process into three distinct phases (beginning, middle, and end of composting) based on metabolic functions [21]. The initial phase was associated with decomposing and utilizing easily degradable organic nutrients, the middle phase with breaking down complex carbon sources, and the final phase with amino acid metabolism, energy production, and signaling processes. These functional activities may provide opportunities for biomarker development to assess compost safety and enhance compost efficiency.

The collective analyses at the São Paulo Zoo Park have provided a comprehensive examination of microbial dynamics within co-composting systems [21, 108, 109]. Such comprehensive investigations serve as valuable models that could improve HEC using CTs, and demonstrate the utility and power of applying integrated multi-omics technologies to advance sustainable waste management.

## Conclusions

Our society generates vast quantities of excrement and other organic materials that are currently managed through largely linear systems but which have the potential to be cycled for improved public health, agricultural productivity and safety, and environmental sustainability. Composting is the microbial biotechnology aimed at driving that transition, and applications of modern microbiome omics and related technologies have vast capacity to support continued advances in composting science and praxis.

CTs and HEC in particular can support improved management of a problematic organic waste material: HE. There are relatively few studies of these important systems employing modern microbiome science technologies, but the studies that have been performed demonstrate the potential of this work [24, 25, 92]. We consider this an opportunity for research that can lead to technological and economic development and have impacts in basic and applied microbiome science. Expanded use of CTs and HE composting across the globe will also rely on overcoming social, cultural, political, and economic barriers: a challenge that could last multiple human life spans [110].

Recognizing the importance of recycling HE and reducing landfill use, biosolids have been increasingly utilized in agriculture and restoration over the past five decades. However, concerns about toxic substances, like PFAS, due to industrial waste contamination highlight the limitations of biosolids. Composting HE before mixing with other waste can enhance safety, and microbiome technologies are crucial for advancing this approach [111].

Linear waste management systems are unsustainable, and a shift toward nutrient cycling, reduced water and energy consumption, and mitigation of pathogen and toxin exposure is urgently needed. Deeper understanding of the activities of microbial communities to degrade toxins and reduce pathogens can lead to targeted inoculation development, biomarker discovery, and bioengineering applications with economic potential. By leveraging microbiome science technologies, numerous stakeholders—including compost managers, researchers, policymakers, for-profit businesses, non-profit entities, and humanitarian aid groups striving to achieve the United Nations’ Sustainable Development Goal 6—can spearhead the development and adoption of more sustainable waste management practices.

## Supplementary Material

Supplementary_Table_1-Compost_Experiments_wrae228

Supplementary_Table_1-caption_wrae228

## Data Availability

Data sharing is not applicable to this article as no datasets were generated or analyzed during the current study.
