# Supplementary material for: Microbiome science of human excrement composting
Source: ISME J. 2024 Nov 9;18(1):wrae228. doi: 10.1093/ismejo/wrae228 (PMC11631093; doi:10.1093/ismejo/wrae228)
Supplement: Supplementary_Table_1-caption_wrae228 [file supplementary_table_1-caption_wrae228.docx]

Supplementary Table 1. Overview of the specific technologies used in the studies reviewed.
